# Elevated plasma EDA fibronectin in primary myelofibrosis is determined by high allele burden of *JAK2*V617F mutation and strongly predicts splenomegaly progression

**DOI:** 10.3389/fonc.2022.987643

**Published:** 2022-09-21

**Authors:** Alessandro Malara, Cristian Gruppi, Margherita Massa, Maria Enrica Tira, Vittorio Rosti, Alessandra Balduini, Giovanni Barosi

**Affiliations:** ^1^ Department of Molecular Medicine, University of Pavia, Pavia, Italy; ^2^ Center for the Study of Myelofibrosis, Laboratory of Biochemistry, Biotechnology and Advanced Diagnostics, IRCCS Policlinico S. Matteo Foundation, Pavia, Italy; ^3^ Department of Biology and Biotechnology “Lazzaro Spallanzani”, University of Pavia, Pavia, Italy

**Keywords:** primary myelofibrosis, splenomegaly, neoangiogenesis, extra domain A, fibronectin

## Abstract

In primary myelofibrosis, extra-domain A fibronectin (EDA-FN), the result of alternative splicing of FN gene, sustains megakaryocyte proliferation and confers a pro-inflammatory phenotype to bone marrow cell niches. In this work we assessed the levels of circulating EDA-FN in plasma samples of 122 patients with primary myelofibrosis. Patients with a homozygous JAK2V617F genotype displayed the higher level of plasma EDA-FN. Increased EDA-FN levels were associated with anemia, elevated high-sensitivity C-reactive protein, bone marrow fibrosis and splanchnic vein thrombosis at diagnosis. While no correlation was observed with CD34+ hematopoietic stem cell mobilization, elevated blood level of EDA-FN at diagnosis was a predictor of large splenomegaly (over 10 cm from the left costal margin) outcome. Thus, EDA-FN expression in primary myelofibrosis may represent the first marker of disease progression, and a novel target to treat splenomegaly.

## Introduction

Fibronectin (FN) mRNA in humans has three alternative splicing sites termed Extra Domain A (EDA), Extra Domain B (EDB) and Type III Homologies Connecting Segment (IIICS), that allow the generation of several FN glycoprotein isoforms (1, 2). FN in blood lacks EDA and EDB segments, while tissue FN contains varying amounts of both Extra Domain segments and is assembled as fibrils in the extracellular matrix (2, 3). EDA-FN is involved in angiogenesis and morphogenesis in embryos (1) but it is largely absent in normal adult tissues. EDA-FN becomes upregulated by stress signals derived from extracellular matrix components and cytokines during wound repair, tissue injury, inflammation, fibrosis and cancer (4–8). In solid cancers there is compelling evidence that EDA-FN upregulation is strictly related with tumor progression and metastasis (9–11). It has been recently demonstrated that EDA-FN is measurable in plasma, and plasma EDA-FN is becoming a useful marker of several pathological processes (12, 13).

Primary myelofibrosis (PMF) is a clonal myeloproliferative neoplasm (MPN) characterized by progressive bone marrow (BM) fibrosis, higher numbers of circulating CD34+ progenitor cells, splenomegaly, cytopenias, and risk of leukemic transformation. Recently, we showed that, in PMF, EDA-FN sustains the proliferation of megakaryocytes and the inflammatory bone marrow microenvironment through Toll Like Receptor-4 binding and cytokine release. Most importantly, we proved that circulating plasma EDA-FN correlates with the extent of BM fibrosis in patients with PMF (14).

The purpose of this study was to define the plasma levels of EDA-FN in a large, well-characterized, cohort of PMF patients. The goal was to uncover the determinants of plasma levels of EDA-FN, and to establish whether increased levels of EDA-FN could predict disease outcomes such as clinical-hematological progression, thrombosis, blast transformation and death.

## Materials and methods

### Patient samples

Plasma samples and health care data of patients with PMF were collected from the database of the Centre for the Study of Myelofibrosis at the IRCCS Policlinico S. Matteo Foundation in Pavia, following informed consent of the subjects. In this study the exclusion criteria were as follows: previous treatment with disease-modifying drugs at any time before or on the date of base-cohort entry, splenectomy, hematopoietic stem cells transplantation, comorbidities such as acute inflammatory diseases, autoimmune diseases, other neoplasms, and severe liver or renal dysfunction. The local Ethical Committee approved the study (Protocol P-20170009376, 01/16/2020).

### ELISA assay

Plasma samples were collected as previously described (14). All plasma samples were immediately stored at -80°C until analysis. Total plasma FN was quantified with a commercially available enzyme-linked immunosorbent assay (ELISA) kit according to manufacturer instructions (Thermo Fischer Scientific, Catalog number HFN1, Milan, Italy). Plasma EDA-FN was measured with an ELISA previously developed and validated in our laboratory (14).

### Immunofluorescence

Spleen specimens were obtained from patients with PMF (N=3; JAK2V617F mutated) that were submitted to splenectomy during disease evolution; while spleen specimens from healthy controls (N=2) were collected in emergency splenectomy secondary to trauma.

Frozen sections were fixed for 20 minutes in paraformaldehyde 4% solution, washed with phosphate-buffered saline (PBS), and blocked with Bovine Serum Albumin (BSA) (Sigma-Aldrich, Milan, Italy) 2% solution for 30 minutes. Sections were then incubated with a blocking solution (5% goat serum, 2% BSA and 0.1% glycine in PBS) for 1 hour. Mouse anti human EDA-FN (clone IST-9, Abcam, Cambridge, UK) and rabbit anti human CD34 (clone EP373Y, Abcam, Cambridge, UK), both diluted 1:100, were incubated overnight at 4°C in washing buffer (0.2% BSA, 0.1% Tween 20 in PBS). Secondary antibodies were goat anti rabbit Alexa Fluor 488 and goat anti-mouse Alexa Fluor 594 (Life Technologies, Milan, Italy). Nuclei were counterstained with Hoechst 33258 (100 ng/mL in PBS). Sections were mounted with micro-cover glasses using Prolong Antifade Reagent (Life Technologies, Milan, Italy). Images were acquired using an Olympus BX51 fluorescence microscope (Olympus Deutschland GmbH, Hamburg, Germany) equipped with a 10X/0.30 Olympus UplanF1 objectives or TCS SP8 confocal laser scanning microscope (Leica, Heidelberg, Germany). Quantitative analysis of EDA-FN staining on spleen sections was obtained from digital microscope images using ImageJ software (NIH) (4). The immunoreactivity was expressed as the surface area of EDA-FN antibody staining normalized on total surface. CD34+ vessels were counted in at least ten images per stained spleen section and expressed as number per mm^2^ of surface. For the quantification of EDA-FN colocalization with CD34+ vessels, the percentages of CD34+/EDA-FN+ vessels were calculated on total CD34+ vessels.

### Statistics

Mann Whitney U test was used to compare differences in EDA-FN concentrations in plasma samples between subjects. Time of progression to large splenomegaly curves were drawn using the Kaplan–Meier procedure. Two Tailed T Test was used to analyze EDA-FN distribution in spleen sections and values are expressed as mean ± SD. A P value of ≤ 0.05 was considered statistically significant throughout analyses. Data were analyzed using STATISTICA software (DELL, release 13.1) and GraphPad Prism (Version 6.0c).

## Results

We conducted a cross-sectional evaluation of plasma EDA-FN in 122 patients with PMF: of these, 63 (52%) were assayed at diagnosis, and 59 (48%) after diagnosis, with a median time of 106 months (range, 13-289 months) from diagnosis to results. All the patients entering the database had the diagnosis of PMF established upon BM biopsy examination according to the most recent WHO criteria. The demographic, clinical, biological, and molecular variables, as well as plasma EDA-FN concentrations, of the analyzed population at and after diagnosis are listed in **Table 1**.

**Table 1:** Demographic, clinical features and plasma EDA-FN concentrations of patients with primary myelofibrosis (PMF) at the time of plasma samples collection used for EDA-FN quantification.

	Total population	Patients at diagnosis	Patients after diagnosis
**Number**	122	63	59
**Males, number (percent)**	63 (51.6)	29 (46)	34 (57.6)
**Age, years; median (range)**	49 (27-80)	47 (27-80)	54 (30-79)
**Hemoglobin, g/dL, median (range)**	12.9 (6.9-17.8)	13.7 (7- 17.8)	11.7 (6.9-15.3)
**White-blood cell count, x10^9^/L, median (range)**	7.7 (1.4-25,1)	7.6 (1.9-29)	7.9 (1.4-29)
**Immature myeloid cells in peripheral blood, percent, median (range)**	0 (0-16)	0 (0-6)	4 (0-13)
**Blasts in peripheral blood, percent, median (range)**	0 (0-6)	0 (0-2)	0 (0-6)
**Platelet count, x10^9^/L, median (range)**	344 (48-1184)	458 (48-1184)	268 (64-1042)
Spleen index, cm^2^, median (range)*	120 (90-665)	100 (90-350)	140 (90-665)
**CD34+ blood cells, x10^6^/L, median (range)**	10.9 (0.6-698)	6.2 (0.75-92.2)	30.3 (1.2-698)
**LDH, times x ULN, median (range)§**	1.18 (0.48-5.9)	1.01 (0.5-4.9)	1.7 (0.48-5.9)
BM fibrosis grade 0-1, number (percent)**	48 (64)	39 (62)	9 (75)
**BM fibrosis grade 2-3, number (percent)**	27 (36)	24 (38)	3 (25)
*JAK2*V617F mutation, number (percent)***	74 (63)	43 (68)	31 (56)
***CALR* mutation, number (percent)**	30 (25)	18 (28)	12 (22)
***MPL* mutation, number (percent)**	5 (4)	0 (0)	5 (9)
***Triple negative* genotype, number (percent)**	9 (8)	2 (4)	7 (13)
***Plasma EDA-FN in μg/mL* ± SD*, mean, median (range)***	5.14 ± 4.09, 3.91 (0.42 - 21.46)	5.9 ± 4.74, 4.38 (0.42 - 21.46)	5.2 ± 4.3, 3.65 (0.65-20.10)

*Spleen index is the product of the longitudinal by the transversal spleen axis, the latter defined as the maximal width of the organ. Splenectomized patients were excluded from the study. Non-palpable spleens were set at 90 cm^2^.

§ ULN = upper limit of normal.

** Bone marrow examination was available in 75 patients.

*** Driver mutations genotype was available in 118 patients.

**Figure 1:**
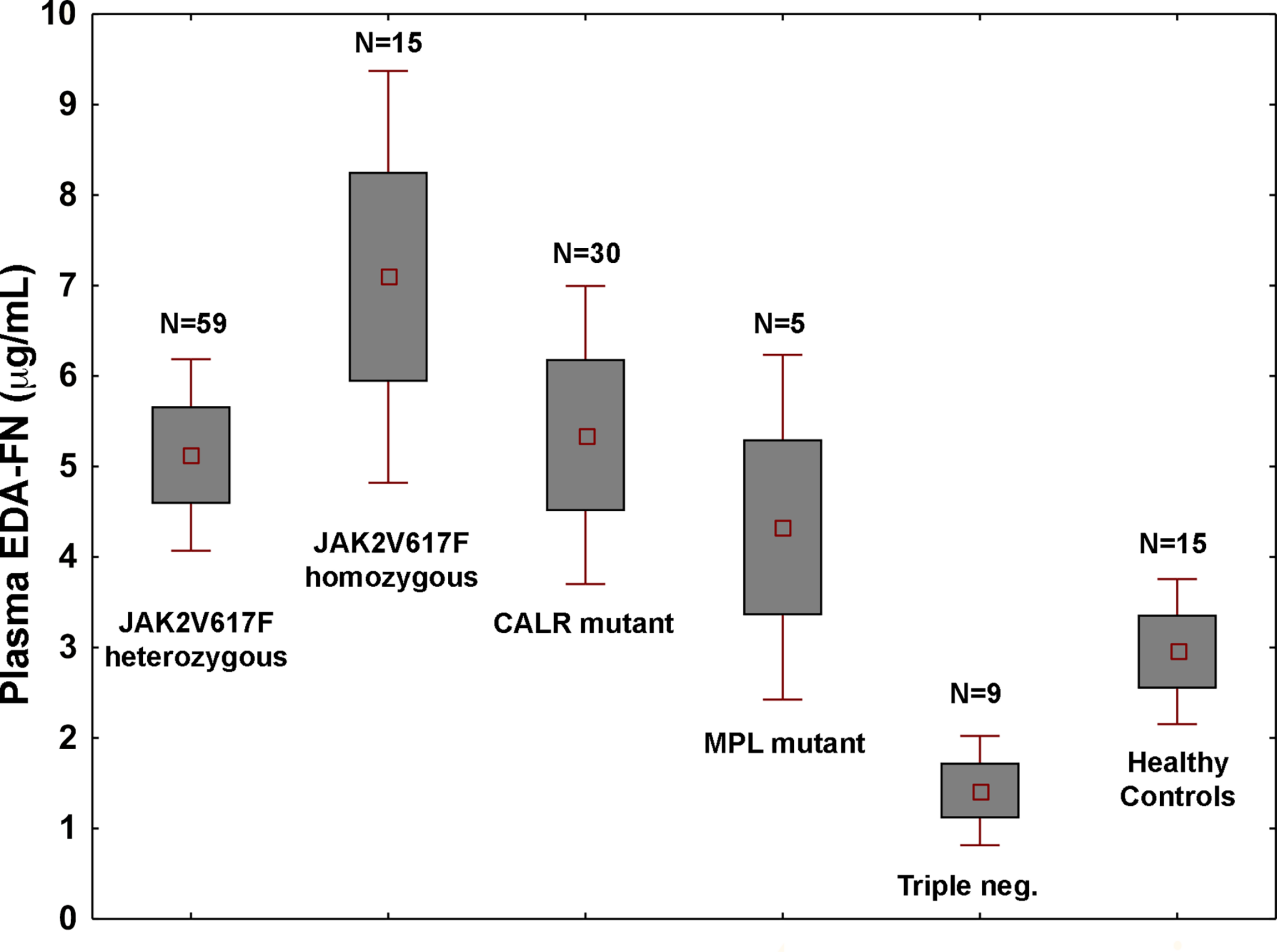
Plasma EDA-FN levels are determined by high allele burden of *JAK2*V617F mutation. Plasma EDA-FN levels (mean ± 1.96 standard error) of subjects with PMF stratified according to the driver mutations genotypes. *JAK2*V617F mutants with >50% allele burden (homozygous genotype) had the highest mean EDA-FN plasma concentration (N=15, mean value = 6.73 μg/mL, range, 2.16 - 19.16). The mean value of EDA-FN in homozygous *JAK2*V617F genotype was higher than in other genotypes together (N=103, mean value = 4.73 μg/mL, P=0.015) and in heterozygous *JAK2*V617F genotype (N=59, mean value = 4.85 μg/mL; P=0.036). Triple negative subjects had the lowest plasma EDA-FN concentration (N=9; mean value = 1.29 μg/mL; range, 0.75 to 6.28); their value was not different from those of healthy controls and was significantly lower than those with *MPL* mutation (N=5, mean value = 5.11 μg/mL, range, 2.57 to 10.82; P=0.016) and *CALR* mutations (N=30; mean value = 5.65 μg/mL, range, 0.65 to 20.11; P=0.039).

Plasma EDA-FN concentrations ranged from 0.42 to 21.46 μg/mL with an average value of 5.14 ± 4.09 μg/mL standard deviation (SD), and a median concentration of 3.91 μg/mL. Plasma EDA-FN metrics in 15 healthy controls (9 Female, 6 Male; Mean Age: 45.6 years) ranged from 0.85 to 5.32 μg/mL with an average concentration of 2.96 ± 1.47 μg/mL (SD), and a median value of 3.05 μg/mL. Median EDA-FN concentration was statistically different among patients with PMF and healthy controls (P=0.036) (**Figure 1**). Assay-specific cut-off value of 5.30 μg/mL for plasma EDA-FN was taken as the upper end of healthy controls (upper 95^th^ centile).

In the whole population of PMF patients, the plasma EDA-FN levels were not correlated with age (r=-0.05; P=0.62) and were not different in males compared to females (mean, 5.11 vs. 5.47 μg/mL; P=0.67). On the contrary, plasma EDA-FN levels were significantly different in patients with different genotypes of the three driver mutations (P=0.05) (**Figure 1**). The difference was driven by very high levels of EDA-FN in subjects with *JAK2*V617F with *≥ a* 50% allele burden (N=15; mean, 6.73 μg/mL, range, 2.16 to 19.16 μg/mL).

At correlation analysis (**Table 2**), EDA-FN plasma levels were not associated with the majority of clinical-hematological variables that represented the myeloproliferative characteristics of the disease, i.e. white blood cell count (r=-0.06; P= 0.49), platelet count (r=-0.13; P=0.20), number of circulating blasts (r=-0.26; P=0.30), LDH plasma concentration (r=-0.07; P=0.63), serum cholesterol (r=-0.23; P=0.20), or absolute monocyte count (r=-0.04; P=0.68). While EDA-FN plasma levels were inversely correlated with hemoglobin (r=-0.21; P=0.025), and directly with spleen size (r=0.27; P=0.003) (**Table 2**). Since the primary cause of splenomegaly in PMF is extramedullary hematopoiesis (EMH) resulting from abnormal mobilization of CD34+ hematopoietic stem/progenitor cells (HSPCs), we analyzed whether EDA-FN plasma levels were associated with circulating CD34+ HSPC frequency. No such a correlation was revealed (r=-0.09; P= 0.35).

**Table 2:** Correlation of EDA-FN plasma levels with demographic, clinical and pathological characteristics of PMF patients. P = p value.

Clinical Parameter	P
Age	0.62
Sex	0.67
Genotype	0.05
White Blood Cell Count	0.49
Monocyte Count	0.68
Platelet Count	0.20
Degree of bone marrow fibrosis	**0.006**
Circulating CD34 progenitor cells	0.35
Circulating Blasts	0.30
LDH concentration (plasma)	0.63
High sensitivity-C reactive protein (hs-CRP)	**0.012**
Cholesterol (serum)	0.20
Hemoglobin	**0.025**
Spleen size	**0.003**

The bold values represent the statistically significant values.

EDA-FN plasma levels resulted significantly correlated with the degree of BM fibrosis (Kruskal-Wallis test, P=0.006) and with the plasma levels of the inflammatory marker high sensitivity-C reactive protein (hs-CRP) (r=0.26; P=0.012).

A splanchnic vein thrombosis (SVT) was coincidentally diagnosed in 10 out of 40 (25%) patients diagnosed with pre-fibrotic MF (BM fibrosis grade 0 or 1). EDA-FN levels were higher in patients with SVT compared to those without SVT (mean, 7.68 vs. 4.08 μg/mL; P=0.044).

**Figure 2:**
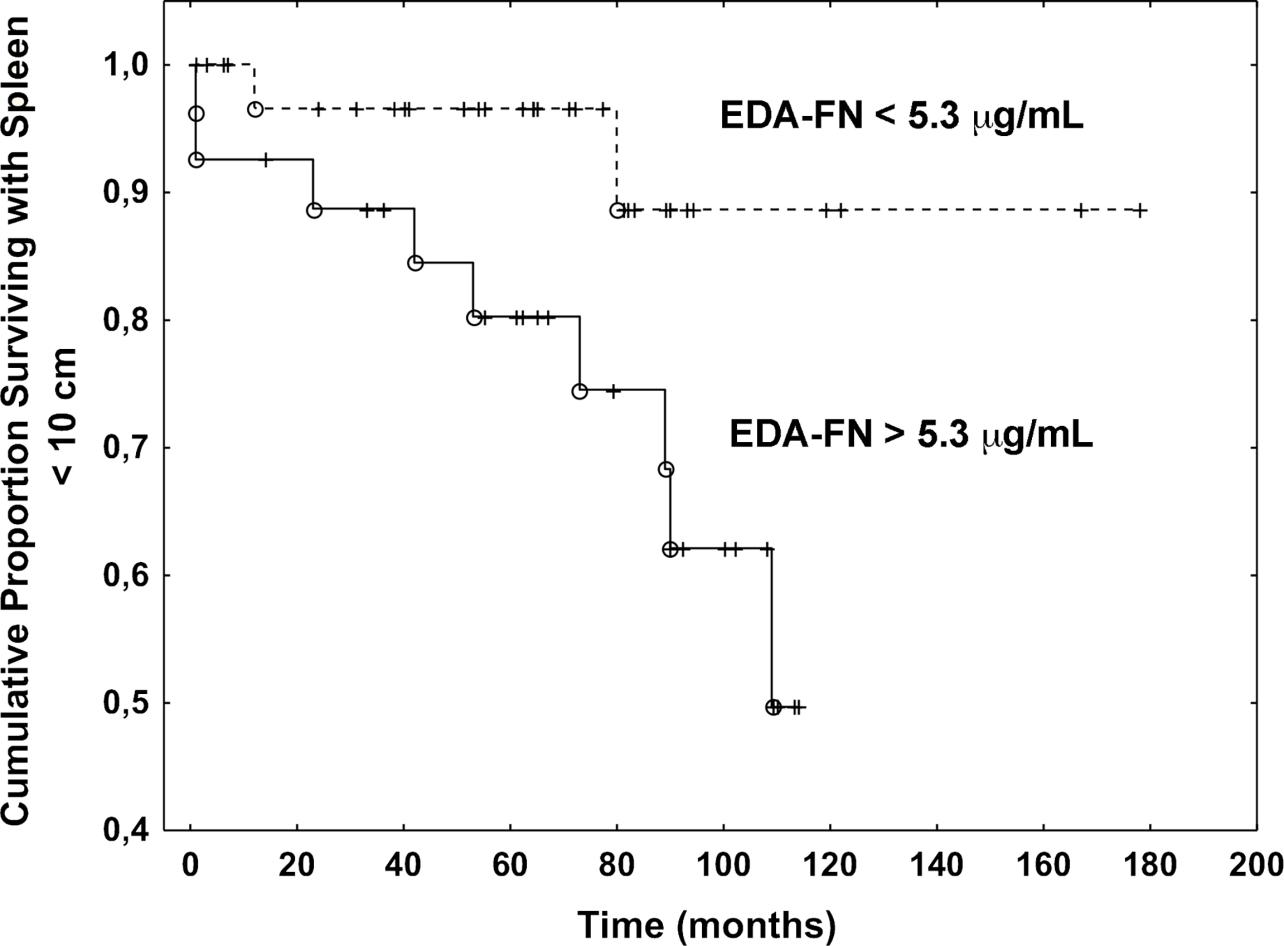
Plasma EDA-FN levels predict splenomegaly progression. Kaplan Meier analysis of the progression to large splenomegaly in patients with EDA-FN lower and greater than the cut-off value of 5.3 μg/mL. The difference was statistically significant (P=0.027).

To test whether high concentrations of plasma EDA-FN might predict the disease progression, we estimated several endpoints using Cox regression models (**Table 3**). In the univariate analysis, the hazard ratio (HR) in patients with elevated EDA-FN group as compared to EDA-FN normal group was not different for the development of severe anemia (hemoglobin less than 10 g/dL; HR, 1.25; 95% CI, 0.46-3.33; P=0.66), leukocytosis (white blood cell count higher than 12 x 10^9^/L; HR, 3.84; 95% CI, 0.55-20; P= 0.11), thrombocytopenia (platelet count lower than 150 x 10^9^/L; HR, 0.91; 95% CI, 0.26-4.51; P=0.92), and leukopenia (white blood cell count lower than 4 x 10^9^/L; HR, 1.08; 95% CI, 0.15-8.3; P=0.67). While, there was a significant difference in the likelihood of developing large splenomegaly (over 10 cm from the costal margin): patients with elevated EDA-FN plasma concentrations at diagnosis were more than five times as likely to progress to large splenomegaly with respect to those with normal levels of EDA-FN (HR, 5.53; 95% CI, 1.21-25.64; P=0.027) (**Figure 2**). Most of the patients with a spleen smaller than 10 cm from the costal margin and EDA-FN levels equal or lower than 5.3 μg/mL maintain a smaller spleen for several years, while those with EDA-FN higher than 5.3 μg/ml do not. We further investigated the relevance of elevated levels of EDA FN on the prediction of splenomegaly outcome by testing EDA-FN with other potential predictor variables that were selected based on previous studies and clinical meaningfulness, and available at least in 90% of patients. Besides EDA-FN, three variables at diagnosis were significantly correlated with splenomegaly progression: WBC >12x10^9^/L (HR = 3.73; 95% CI, 1.09-12.82; P=0.036), CD34+ blood cells > 50 x10^6^/L (HR = 11.11; 95% CI, 1.95-58.82; P=0.006), and spleen size (HR = 1.03; 95% CI, 1.01-1.04; P=0.001). At the multi-variable analysis, only EDA-FN maintained an independent prediction value for splenomegaly progression (HR= 5.88, 95% CI, 1.65-52.63; P=0.050).

**Table 3:** Univariate Cox regression analysis of EDA-FN plasma levels with clinical endpoints in PMF patients with elevated EDA-FN group as compared to EDA-FN normal group. HR = Hazard Ratio, P = p value.

Variable	HR	CI 95%	P
Severe Anemia (Hemoglobin less than 10 g/dL)	1.25	0.46-3.33	0.66
Leukocytosis (White blood cell count higher than 12 x 10^9^/L)	3.84	0.55-20	0.11
Thrombocytopenia (Platelet count lower than 150 x 10^9^/L)	0.91	0.26-4.51	0.92
Leukopenia (White blood cell count lower than 4 x 109/L)	1.08	0.15-8.3	0.67
Splenomegaly (Development of large splenomegaly, over 10 cm from the costal margin)	5.53	1.21-25.64	**0.027**
Thrombosis	1.02	0.32-3.22	0.98
Blast transformation	0.88	0.17-4.54	0.87
Death	0.84	0.16-4.34	0.83

The bold values represent the statistically significant values.

No statistically significant association was found between elevated levels of EDA-FN and occurrence of thrombosis (HR = 1.02; 95% CI, 0.32-3.22; P=0.98), blast transformation (HR = 0.88; 95% CI, 0.17-4.54; P=0.87), or death (HR = 0.84; 95% CI, 0.16-4.34; P=0.83).

**Figure 3:**
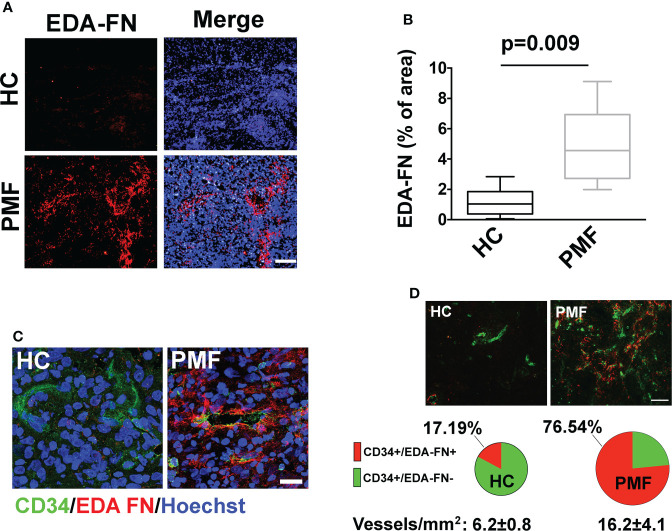
EDA-FN is increased in spleen of PMF patients. **(A)** Confocal microscopy analysis of FN containing EDA segment (Ab IST-9) (red) in spleen biopsies of healthy control (HC) and Primary Myelofibrosis (PMF) patients. Nuclei were stained with Hoechst 33258. Objective 10x, Scale Bar = 100μm. **(B)** EDA-FN immunoreactivity expressed as % of EDA-FN antibody staining area in spleens of HC (N=2) and PMF patients (N=3) normalized on total surface. **(C)** Confocal microscopy analysis of EDA-FN localization (red) in proximity of CD34+ vessels (green) in spleen sections of HC and PMF patient. Hoechst 33258 was used to stain nuclei. Objective 60x, Scale bar = 50 μm. **(D)** Quantification of total CD34+, CD34+/EDA-FN- and CD34+/EDA-FN+ vessels in spleen biopsies of HC (N=2) and PMF patients (N=3). At least 10 fields per sample were analyzed. Objective 20x, Scale bar = 100 μm.

We interpreted the resulting association of EDA-FN plasma levels with anemia, BM fibrosis, hs-CRP, and SVT as reflecting the critical role of EDA-FN in tissue repair, inflammation and vascular disease (4–6). To explain the association with spleen size and splenomegaly progression, we considered the evidence that increased EDA-FN expression in solid tumors is mostly related with tumor angiogenesis and endothelial activation (15–17), and that neo-angiogenesis and endothelial activation are now documented in the spleen of PMF patients (18–22). *JAK2*V617F mutation and endothelial-mesenchymal transition, mechanisms of endothelial activation (15), are present in spleen endothelial cells of PMF patients (19–22). We performed immunofluorescence microscopy analysis on spleen sections of three independent PMF patients carrying the *JAK2*V617F mutation and compared results to two healthy samples. Results demonstrated that, while in healthy controls EDA-FN was almost undetectable, in patient samples EDA-FN was significantly increased (**Figures 3A, B**). In patient spleen sections, high levels of EDA-FN were detected in proximity of the CD34+ vessels (**Figure 3C**). Neo-angiogenesis was evident in spleen biopsies from PMF patients as demonstrated by the increased frequency of CD34+ vessels/mm^2^ of tissue compared to controls (HC 6.2 ± 0.8 vs PMF 16.2 ± 4.1; ***P<0.001). EDA-FN surrounded most of the CD34+ vessels in PMF (76.54%), whereas it was less frequently detected around healthy control CD34+ vessels (17,19%) (**Figure 3D**).

## Discussion

Fibronectin (FN), a multifunctional glycoprotein, has twenty different isoforms. Alternative splicing of the pre-mRNA at three different splice sites (extra domain A (EDA), extra domain B (EDB), and type III homology connecting segment (IIICS)) is crucial for FN protein diversity (1). Circulating plasma FN isoform is soluble, produced by hepatocytes, and lacks both the EDA and EDB segments, while cellular FN isoforms in the extracellular matrix of tissues may contain variable proportions of EDA or EDB or both segments (2). EDA-FN is expressed nearly ubiquitously during embryogenesis (3), while its expressions is barely detected in normal adult tissues but strongly induced in several pathological states (5–8). Previous studies have demonstrated that EDA-FN is implicated in the fibrotic evolution of several organs, mostly skin, lung and liver (5, 23), as well as other pathological processes (24).

PMF is classified as a BCR-ABL1-negative MPN characterized by progressive bone marrow fibrosis, splenomegaly, consequent to extramedullary hematopoiesis, and leukoerythroblastosis (25). We recently showed that, compared to healthy controls, patients with PMF exhibit elevated levels of EDA-FN in plasma and bone marrow biopsies during fibrosis progression highlighting the crucial role of EDA-FN in the disease pathogenesis and its utility as a prognostic biomarker in MPNs (14). In the present study, we evaluated whether plasma levels of EDA-FN correlate with several clinical and biological prognostic markers of MPNs or predict the occurrence of disease-related events in a large well-characterized population of PMF patients. Our results demonstrate that PMF patients, harboring a homozygous JAK2V617F genotype, display the higher plasma EDA-FN concentrations and that elevated blood EDA-FN significantly correlates with anemia, high levels of hs-CRP, grade of BM fibrosis and splanchnic vein thrombosis at diagnosis. No correlation was observed with CD34+ hematopoietic stem cells mobilization. Most importantly, high plasma EDA-FN concentrations at diagnosis predicted large splenomegaly outcome.

In solid tumors, the expression of EDA-FN in vascular endothelial cells is a parameter of cancer-related neo-angiogenesis and a target for cancer therapy or imaging (26, 27). Thus, to explain the association of EDA-FN levels with splenomegaly and spleen size progression in PMF we focused on its well-known role in tumor angiogenesis and endothelial activation (28, 29). Neo-angiogenesis has been reported in the BM of PMF patients, and, consistently, the increased capillary vascular density in the spleen of patients is considered a major determinant for the spleen volume expansion (18). JAK2V617F mutation and endothelial-mesenchymal transition were reported in spleen endothelial cells of PMF patients (19–22). Immunofluorescence analysis, in spleen sections, confirmed a significant increase in the deposition of EDA-FN in PMF patients with respect to healthy controls and a co-localization with splenic endothelial cells.

In conclusion, besides the associations of EDA-FN with BM fibrosis, inflammation, and thrombosis, we unexpectedly found that, in PMF patients, high plasma concentrations of EDA-FN, at diagnosis, predicted large splenomegaly outcome. We hypothesize that EDA-FN could represent a marker of endothelial cell activation and/or neo-angiogenesis in the spleen of patients. Thus, EDA-FN expression in PMF may represent the first marker of vascular alteration of the spleen and a potential novel therapeutic target for splenomegaly in PMF.

## Data availability statement

The raw data supporting the conclusions of this article will be made available by the authors, without undue reservation.

## Ethics statement

The studies involving human participants were reviewed and approved by Ethical Committee of IRCCS San Matteo Foundation. The patients/participants provided their written informed consent to participate in this study.

## Author contributions

AM designed and performed the experiments and wrote the manuscript. CG performed the experiments and edited the manuscript. VR, MM, MT, and GB provided materials and patient samples, analyzed the data, and edited the manuscript. GB performed the statistical analysis and wrote the manuscript. AB supervised the project, conceived the idea, analyzed the data, and wrote the manuscript. All authors contributed to the article and approved the submitted version.

## Funding

This paper was supported by Associazione Italiana per la Ricerca sul Cancro (AIRC IG 2016 18700, AIRC; Milano, Italy) to AB; Italian Ministry of Health (Ricerca Finalizzata Giovani Ricercatori GR-2016-02363136) to AM.

## Conflict of interest

The authors declare that the research was conducted in the absence of any commercial or financial relationships that could be construed as a potential conflict of interest.

The reviewer SM declared a past co-authorship with one of the authors AB to the handling Editor.

## Publisher’s note

All claims expressed in this article are solely those of the authors and do not necessarily represent those of their affiliated organizations, or those of the publisher, the editors and the reviewers. Any product that may be evaluated in this article, or claim that may be made by its manufacturer, is not guaranteed or endorsed by the publisher.
